# Understanding the role of antibody glycosylation through the lens of severe viral and bacterial diseases

**DOI:** 10.1093/glycob/cwaa018

**Published:** 2020-02-26

**Authors:** Edward B Irvine, Galit Alter

**Affiliations:** 2 Ragon Institute of MGH, MIT, and Harvard, Cambridge, MA, USA; 3 Department of Immunology and Infectious Diseases, Harvard T.H. Chan School of Public Health, Boston, MA, USA

**Keywords:** antibody, Fc, glycosylation, infectious disease, humoral immunity

## Abstract

Abundant evidence points to a critical role for antibodies in protection and pathology across infectious diseases. While the antibody variable domain facilitates antibody binding and the blockade of infection, the constant domain (Fc) mediates cross talk with the innate immune system. The biological activity of the Fc region is controlled genetically via class switch recombination, resulting in the selection of distinct antibody isotypes and subclasses. However, a second modification is made to all antibodies, via post-translational changes in antibody glycosylation. Studies from autoimmunity and oncology have established the role of immunoglobulin G (IgG) Fc glycosylation as a key regulator of humoral immune activity. However, a growing body of literature, exploring IgG Fc glycosylation through the lens of infectious diseases, points to the role of inflammation in shaping Fc-glycan profiles, the remarkable immune plasticity in antibody glycosylation across pathogen-exposed populations, the canonical and noncanonical functions of glycans and the existence of antigen-specific control over antibody Fc glycosylation. Ultimately, this work provides critical new insights into the functional roles for antibody glycosylation as well as lays the foundation for leveraging antibody glycosylation to drive prevention or control across diseases.

## Introduction

Antibodies are a critical component of the protective immune response across many infectious diseases and represent primary correlates of protection for the majority of approved vaccines (Mason et al. 1973; Faden et al. 1990; Jack et al. 1999; Lee et al. 2003; Frazer 2007; Plotkin 2010; Achkar and Casadevall 2013; Wheatley and Kent 2015). Antibodies are composed of two functional domains, the variable domain (F(ab′)_2_) and the constant domain (Fc). The F(ab′)_2_ is responsible for the recognition of antigen and, accordingly, can contribute to pathogen blockade or neutralization, whereas the Fc region interacts with a variety of different Fc receptors (FcRs), as well complement proteins, to link the exquisite specificity of the adaptive immune system to the powerful effector functions and biological activity of the innate immune system (Spiegelberg 1989). The biological activity of the Fc region is tuned at the genomic level by the selection of antibody isotype and subclass and at the post-translational level by glycosylation. Although less appreciated than antibody isotype and subclass selection, accumulating data across diseases points to the critical importance of antibody glycosylation as a regulator of antibody stability, half-life, secretion, immunogenicity and function (Arnold et al. 2007; Pincetic et al. 2014).

While many of the biological effects of antibody glycosylation were discovered or investigated in detail in the context of autoimmune and oncological diseases, infectious diseases have pointed to the remarkable immunologic control over antibody glycosylation—how it is shaped by inflammation and, more importantly, how it may be tractably controlled to direct immune function. Because the capture and analysis of known pathogen-specific antibodies is possible, which is often difficult in autoimmune conditions and cancer, we can observe how changes occur in an infection-directed manner and how they track with altered antibody effector function. Thus, these models help elucidate the role of antigen-specific antibody glycosylation control, define the role of these antibodies in protection or pathology and point to novel therapeutic opportunities to tune immunity via antibody glycosylation.

In this review, we will discuss antibody Fc glycosylation in the context of infectious diseases. We will first cover the essentials of antibody Fc glycosylation, discussing how immunoglobulin G (IgG) Fc glycosylation selectively modulates the immune response through interactions with distinct antibody sensors. Then, using a few well-characterized viral and bacterial diseases as models, we will explore the plethora of ways in which IgG Fc glycosylation is tuned by, and may direct the immune response to infection. Finally, we will discuss the prospects for leveraging antibody Fc glycosylation to combat infectious diseases.

## Antibody Fc domain is modified during infection

### Antibody isotype and subclass selection

During infection, the biological activity of the Fc region is first tuned at the genomic level via the selection of antibody isotype and subclass. In humans, there are five distinct antibody isotypes: IgM, IgD, IgE, IgA and IgG (Franklin 1975). Furthermore, within the IgG and IgA isotypes, there are additional subclasses: IgG1—IgG4 and IgA1—IgA2, respectively (Spiegelberg 1989). Each antibody isotype and subclass play distinct roles in the immune response based on the kinetics by which they appear, as well as their ability to interact both with the pathogen and the innate immune system (Spiegelberg 1989).

### Antibody Fc glycosylation

**Fig. 1:**
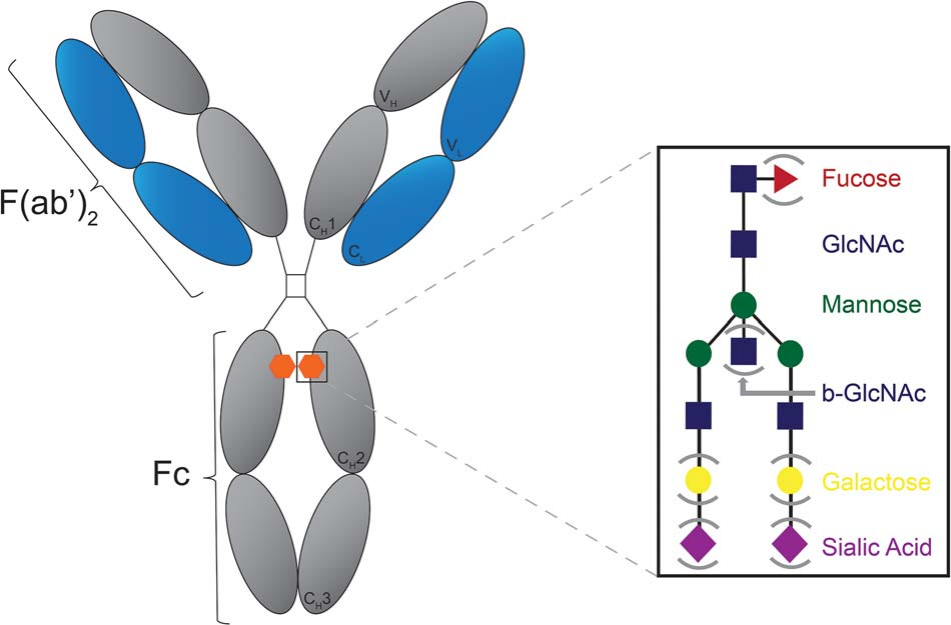
Structure of IgG and the IgG N-linked glycan. IgG molecules have a single N-linked glycosylation site at asparagine 297 of each heavy chain. The base glycan structure is pictured, with each of the four variably added glycan moieties present in parentheses.

Beyond isotype selection, every antibody Fc is also post-translationally modified via the addition of *N*-glycans at specific asparagine residues on the antibody heavy chain (Arnold et al. 2007). All IgG molecules bear a single N-linked glycosylation site at asparagine 297 (N297) of each heavy chain (Figure 1; Arnold et al. 2007).

IgG Fc glycosylation is always N-linked, biantennary and has a restricted repertoire of glycans that can be added compared to other N-glycosylated proteins (Arnold et al. 2007; Varki et al. 2015). The *N*-glycan is first added to the IgG Fc in the endoplasmic reticulum (ER) of antibody secreting B cells (plasmablasts or plasma cells), where a single homogenous high-mannose structure is added to the growing antibody polypeptide chain cotranslationally (Varki et al. 2015). Once IgG transits from the ER to the Golgi, it encounters a sequence of glycosidases and glycosyltransferases that modify the highly mannosylated glycan structure in a highly organized manner, trimming and extending the glycan to form the classical biantennary “core glycan” structure (Varki et al. 2015). The core glycan is composed of two consecutive *N*-acetylglucosamine (GlcNAc) molecules, followed by a mannose, followed by two additional mannose antennae, each with a single GlcNAc attached (Varki et al. 2015). The core glycan is then further modified sequentially by a set of four glycosyltransferases. Fucosyltransferase 8 may add a core fucose within the medial Golgi (fucosylation). Next, within the trans Golgi, beta-1,4-galactosyltransferase 1 may add one or two galactoses to the antennary GlcNAcs (galatosylation), MGAT3 may add a bisecting GlcNAc (bisection) and ST6 beta-galactoside alpha-2,6-sialyltransferase 1 may add one sialic acid to each galactose structure present (sialylation) (Figure 1; Varki et al. 2015).

Furthermore, potential steric limitations exist (Nishima et al. 2012), which may limit the abundance of certain glycoforms. Nevertheless, despite the highly ordered, restrictive nature of IgG Fc glycosylation, variation in fucosylation, galactosylation, bisection and sialylation of the N-linked glycan means that 36 different glycan structures may theoretically be created (Jennewein and Alter 2017). Thus, while termed the constant domain, the IgG Fc region contains an immense level of heterogeneity, providing the immune system an opportunity to tune the Fc to acquire precise control of antibody activity during an immune response.

Conversely, IgM (5), IgD (3), IgE (7), IgA1 (2) and IgA2 (5) each have the indicated number of N-linked glycosylation sites on their respective heavy chains that are modified by more complex glycans (Arnold et al. 2007). Furthermore, IgD and IgA1 each possess multiple O-linked glycosylation sites (Arnold et al. 2007). While the large number of Fc glycosylation sites on these additional isotypes provides immense potential for functional heterogeneity, the majority of infection-associated immune profiling has focused on deconvoluting IgG Fc glycosylation changes, which will therefore be the topic of this review.

### IgG Fc glycosylation regulates antibody function

A diverse array of FcRs and complement proteins, which we refer to as antibody sensors, has evolved in mammals over time to combat infection and regulate immunity. These sensors each interact with different subgroups of antibodies—depending on the molecular characteristics of the antibody Fc region—and trigger distinct signaling pathways to modulate function and inflammation, many of which are critical in the antipathogen response. For example, mice lacking Fcγ receptors demonstrate increased bacterial burden and reduced survival following *Mycobacterium tuberculosis* (*Mtb*) challenge (Maglione et al. 2008), whereas the elimination of complement results in increased pathology and mortality across several viral infections (Mehlhop et al. 2005; O’Brien et al. 2011). Given the importance of glycosylation in shaping complement, Fcγ receptor and C-type lectin receptor binding (Pincetic et al. 2014), the impact of differential IgG Fc glycosylation on antibody signaling through these sensors will be discussed.

The IgG glycan at N297 is nestled between the heavy chains of the antibody Fc-domain, contributing to both the structural integrity and conformation of the Fc region (Krapp et al. 2003; Yamaguchi et al. 2006). Specifically, the presence of an N-linked glycan at this site is believed to maintain the Fc domain in an open conformation, allowing interaction of the IgG Fc region with Fcγ receptors (Krapp et al. 2003), as removal of the Fc-glycan, or the elimination of the asparagine at N297, both diminish Fcγ receptor binding and significantly reduce antibody functionality (Nose and Wigzell 1983; Tao and Morrison 1989; Shields et al. 2001; Arnold et al. 2007; Jefferis 2009). Furthermore, each of the variably added glycan moieties that are added to the Fc-glycan—fucose, bisecting GlcNAc, galactose and sialic acid—has been implicated in modulating antibody sensor affinity and/or antibody function.

#### Fucosylation

Over 90% of IgG in the serum of healthy individuals is core fucosylated in the Fc region (Gudelj et al. 2018). Decades of work in the monoclonal therapeutics community has clearly demonstrated the critical role of a loss of fucosylation as a key determinant of Fc affinity for FcγRIIIa (Sondermann et al. 2000; Shields et al. 2002; Shinkawa et al. 2003; Ferrara et al. 2006, 2011). Specifically, the complete removal of fucose from the IgG Fc increases IgG Fc-affinity to FcγRIIIa by 50-fold (Shinkawa et al. 2003). Given the robust expression of FcγRIIIa on NK cells, activation of FcγRIIIa results in potent NK cell activation (Simmons and Seed 1988). Hence, core afucosylation significantly enhances ADCC (Shields et al. 2002; Shinkawa et al. 2003).

#### Bisection

In healthy adults, approximately 10% of circulating IgG has an Fc bisecting GlcNAc moiety (b-GlcNAc) (Gudelj et al. 2018). Monoclonal therapeutic work has demonstrated that the addition of a b-GlcNAc to the core glycan increases ADCC activity (Shinkawa et al. 2003; Hodoniczky et al. 2005). However, if MGAT3, the glycosyltransferase that adds the b-GlcNAc to the IgG Fc-glycan, adds a b-GlcNAc early in glycan processing, fucosylation can be inhibited (Shinkawa et al. 2003; Nishima et al. 2012; Kurimoto et al. 2014). Thus, the addition of a b-GlcNAc moiety likely affects antibody effector function indirectly by inhibiting the addition of fucose.

#### Galactosylation

In healthy adults, agalactosylated, monogalactosylated and digalactosylated glycan structures account for approximately 35, 35 and 15% of circulating IgG Fc-glycan, respectively (Gudelj et al. 2018). However, patients with active autoimmune and inflammatory diseases shift the balance of these glycan species largely toward an accumulation of agalactosylated IgG (Parekh et al. 1989; Tomana et al. 1992; Trbojević Akmačić et al. 2015; Decker et al. 2016). As a result, agalactosylation is generally regarded as proinflammatory, however, the extent to which these associations represent cause or consequence remains incompletely defined.

Moreover, contradictory data have emerged about the effect of galactosylation on antibody function, as IgG galactosylation has been reported to mediate pro-inflammatory effects, to mediate anti-inflammatory effects, as well as to have no effect on inflammation or innate immunity. For example, galactosylation of the IgG Fc region has been observed to modestly enhance C1q binding and ADCC in vitro (Tsuchiya et al. 1989; Nimmerjahn et al. 2007; Thomann et al. 2016; Peschke et al. 2017). Conversely, galactosylated IgG immune complexes have been demonstrated to dampen C5a-mediated inflammation in mice in an FcγRIIb- and dectin-1-dependent manner in vivo, suggesting that the cooperative signaling of FcγRIIb and dectin-1 results in the blockade of inflammation (Karsten et al. 2012). Finally, the work using platelet-specific monoclonal antibodies demonstrated that the presence or absence of galactose did not impact the in vivo activity of the antibodies in mice (Nimmerjahn et al. 2007). Thus, together, these data indicate that galatosylation may have diverging influences on antibody functionality.

#### Sialylation

In healthy adults, approximately 10% of circulating IgG is Fc sialylated (Gudelj et al. 2018). The functional role of IgG Fc sialylation was first noted in studies focused on dissecting the functional role of IVIg (Kaneko et al. 2006; Anthony, et al. 2008a; Anthony, et al. 2008b; Anthony et al. 2011a, 2011b). Specifically, IVIg was treated with neuraminidase to remove the sialic acid residues, and then, the ability of neuraminidase-treated IVIg to inhibit inflammatory responses was probed in a mouse model of RA (Kaneko et al. 2006). Strikingly, neuraminidase treatment abrogated the protective effect of IVIg normally observed in this model, pointing to the critical role of sialylated IgGs in dampening inflammation (Kaneko et al. 2006). Mechanistically, the anti-inflammatory activity was shown to depend on the binding of sialylated IgG to DC-SIGN, resulting in the ultimate upregulation of the inhibitory FcγRIIb on macrophages, thereby reducing inflammation (Anthony et al. 2011a, 2011b).

However, others have reported the interaction between sialylated IgG and DC-SIGN not to be required for the anti-inflammatory effect of IVIg (Campbell et al. 2014). Moreover, work comparing DC-SIGN affinity for hypersialylated, desialylated, deglycosylated and untreated serum IgG found that DC-SIGN bound each of these different immunoglobulin preparations with very similar affinity (Yu et al. 2013). In short, while increasing evidence points to a role for IgG sialylation in mediating anti-inflammatory activity (Kaneko et al. 2006; Anthony, Nimmerjahn, et al. 2008; Anthony, Wermeling, et al. 2008; Anthony et al. 2011a, 2011b; Schwab et al. 2012; Washburn et al. 2015; Ohmi et al. 2016; Zhang et al. 2016; Bartsch et al. 2018; Bozza et al. 2019), the sialylated IgG:DC-SIGN axis, as well as additional immune functions mediated by IgG Fc sialylation, remains active areas of investigation.

## IgG Fc glycosylation in infectious diseases

While many of the rules governing the impact of IgG Fc glycosylation on the biological activity of antibodies were discovered in the context of monoclonal therapeutic development (Li et al. 2017), autoimmune disease (Kaneko et al. 2006) and cancer (Jefferis 2009), antibody glycosylation is known to play a major role in the response to infectious diseases as well (Zeitlin et al. 2011; Ackerman et al. 2013; Wang et al. 2015, 2017; Lu et al. 2016). Specifically, emerging data across a collection of viral and bacterial diseases suggest that striking changes occur in antibody glycosylation during infection (Moore et al. 2005; Vadrevu et al. 2018). These changes act as both markers of disease state but additionally may functionally contribute to control or exacerbation of disease (Wang et al. 2015, 2017; Lu et al. 2016).

### Viral hepatitis

Hepatitis viruses are the most common causal agent of hepatitis, an inflammation of the liver. Globally, approximately 257 million people were chronically infected with hepatitis B virus (HBV) in 2015 (WHO 2018a). Chronic infection leads to liver fibrosis and, without intervention, can result in cirrhosis and liver cancer (WHO 2018a).

Notably, in the setting of HBV infection, IgG Fc glycosylation profiles track with disease severity. Specifically, a study that compared IgG Fc glycosylation profiles between healthy individuals, individuals with HBV-related cirrhosis and individuals with chronic HBV infection found that the two HBV-exposed populations exhibited decreased IgG Fc galactosylation when compared to healthy controls (Ho et al. 2015). Moreover, reduced IgG Fc galactosylation was positively correlated with the severity of fibrosis. This IgG Fc glycosylation pattern is reminiscent of the IgG Fc glycosylation profiles observed in the setting of a plethora of autoimmune diseases (Parekh et al. 1989; Tomana et al. 1992; Trbojević Akmačić et al. 2015; Decker et al. 2016). Furthermore, antiviral therapy reversed the IgG Fc galactose deficiency in individuals with chronic HBV (Ho et al. 2015), suggesting that in this context, attenuating inflammation caused by chronic viral replication allowed the restoration of baseline IgG glycosylation profiles. Finally, these differences in IgG Fc galactosylation were linked to differences in antibody function, as galactose-deficient IgG in individuals with chronic HBV infection displayed a reduced ability to drive opsonophagocytosis as compared to IgG from healthy individuals, as well as IgG from chronic HBV individuals following antiviral treatment (Ho et al. 2015).

Ultimately, while the reported shifts in IgG Fc glycosylation in the setting of HBV infection likely represent a consequence of the chronic inflammatory nature of hepatitis disease due to persistent viral replication, these data demonstrate clear shifts in bulk- and antigen-specific IgG Fc glycosylation that track with different degrees of disease pathology and may thus hold prognostic value (Table I).

**Table I:** Degree of change in Fc glycosylation

Degree of change in IgG Fc glycosylation	Reference
Fucosylation	
*Tuberculosis*	
~91% fucosylation in ATB individuals compared to ~88% in LTBI individuals in a South African cohort	Lu et al. 2016
~81% fucosylation in ATB individuals compared to ~77% in LTBI individuals in a Texas/Mexico cohort	Lu et al. 2016
*HIV*	
~20% monogalactosylated, fucosylated gp120-specific structures in HIV controllers compared to ~25% in untreated HIV-infected individuals	Ackerman et al. 2013
~5% digalactosylated, fucosylated gp120-specific structures in HIV controllers compared to ~9% in untreated HIV-infected individuals	Ackerman et al. 2013
~89% fucosylation in HIV-positive individuals compared to ~83% in HIV-negative individuals.	Vadrevu et al. 2018
*DENV*	
~16% afucosylated DENV env-specific structures in individuals with DHF compared to ~11% in individuals with DENV fever	Wang et al. 2017
~15% afucosylated DENV env-specific structures in DENV patients with thrombocytopenia compared to ~8% in DENV patients without thrombocytopenia.	Wang et al. 2017
Galactosylation	
*Viral hepatitis*	
40.5% and 39.5% agalactosylated structures in patients with HBV-related liver cirrhosis and chronic hepatitis B, respectively; 33.9% in healthy controls	Ho et al. 2015
*Tuberculosis*	
33% agalactosylated structures in children with TB compared to 26.3% in healthy children	Pilkington et al. 1995
~41% agalactosylated structures in ATB individuals compared to ~22% in LTBI individuals in a South African cohort	Lu et al. 2016
~31% agalactosylated structures in ATB individuals compared to ~20% in LTBI individuals in a Texas/Mexico cohort	Lu et al. 2016
~20% digalactosylated structures in ATB individuals compared to ~32% in LTBI individuals in a South African cohort	Lu et al. 2016
~40% digalactosylated structures in ATB individuals compared to ~52% in LTBI individuals in a Texas/Mexico cohort	Lu et al. 2016
*HIV*	
~36–60% of total neutral oligosaccharides were agalactosylated in HIV-positive individuals compared with 4-17% in HIV-negative individuals	Moore et al. 2005
~35% agalactosylated structures in HIV-positive individuals compared to ~20% in HIV-negative individuals	Vadrevu et al. 2018
~56% fucosylated, agalactosylated structures in HIV controllers; ~22% in individuals with acute HIV; ~12% in healthy controls	Ackerman et al. 2013
*Influenza*	
10.9% increase in influenza-specific galactosylation 21 days post influenza vaccination in a cohort of Caucasian adults	Selman et al. 2012
18.3% increase in influenza-specific galactosylation 14 days post influenza vaccination in a cohort of African children	Selman et al. 2012
Sialylation	
*Tuberculosis*	
~12% sialylated structures in ATB individuals compared to ~20% in LTBI individuals in a South African cohort	Lu et al. 2016
~33% sialylated structures in ATB individuals compared to ~40% in LTBI individuals in a Texas/Mexico cohort	Lu et al. 2016
*HIV*	
~10% disialylated structures in HIV-positive individuals compared to ~16% in HIV-negative individuals	Vadrevu et al. 2018
~18% gp120-specific sialylated structures in neutralizers compared to ~11% in non-neutralizers	Lofano et al. 2018
*Influenza*	
8.2% increase in influenza-specific sialylated structures 21 days post influenza vaccination in a cohort of Caucasian adults	Selman et al. 2012
10.3% increase in influenza-specific sialylated structures 14 days post influenza vaccination in a cohort of African children	Selman et al. 2012
~10% increase in HA-specific sialylated structures 7 days post influenza vaccination	Wang et al. 2015

Chart indicates the approximate magnitude difference in IgG glycosylation patterns reported in the different infectious disease infection and vaccination cohorts cited. ATB, active tuberculosis disease; LTBI, latent tuberculosis infection; HIV, human immunodeficiency virus; DENV, Dengue virus; DHF, dengue hemorrhagic fever; HBV, hepatitis B virus; HA, hemagglutinin.

### Tuberculosis

With a quarter of the world infected with *M. tuberculosis* (*Mtb*), and 1.7 million deaths related to these bacteria alone in 2017, *Mtb* is the top infectious disease killer globally (WHO 2019a). However, infection presents on a spectrum between two clinical states: a controlled latent tuberculosis infection (LTBI), with an absence of disease symptoms, and active tuberculosis disease (ATB), marked by pulmonary and potentially disseminated disease (Pai et al. 2016). Though asymptomatic, LTBI individuals carry a 10% lifetime risk of progressing to ATB (Pai et al. 2016). While diagnostics exist to assess TB exposure, no current immune-based diagnostics exist to classify *Mtb*-exposed individuals into those with LTBI or ATB, and more importantly, no defined correlate of protection has been established for *Mtb* control (Pai et al. 2016).

Significant differences in antibody glycosylation have been observed in the setting of tuberculosis disease (Rademacher et al. 1988; Parekh et al. 1989; Pilkington et al. 1995; Lu et al. 2016; Kumagai et al. 2019). In humans, subjects with ATB exhibit an increase in proinflammatory IgG glycans, marked by high levels of agalactosylated and asialylated IgG (Rademacher et al. 1988; Parekh et al. 1989; Pilkington et al. 1995). This glycosylation pattern is consistent with that of severe HBV infection as described above (Ho et al. 2015).

Conversely, bulk and purified protein derivative-specific IgG from LTBI individuals had increased galactosylation and higher levels of sialylation as compared to IgG from ATB individuals (Lu et al. 2016). These differences between ATB and LTBI individuals are likely driven by the drastically different inflammatory states that define each clinical group. ATB individuals are characterized by high bacterial loads that are uncontrolled within granuloma structures in the lung (Pai et al. 2016). This failure to control infection results in protracted inflammation and clinical pathology (Pai et al. 2016). In contrast, LTBI subjects, although *Mtb* exposed, contain *Mtb* within intact granulomas (Pai et al. 2016). Transcriptional analyses have corroborated the inflamed nature of ATB disease, as type I interferon-driven blood transcriptional signatures of ATB have been shown to distinguish this population from LTBI and healthy individuals (Berry et al. 2010; Singhania et al. 2018). Thus, the enrichment in proinflammatory IgG glycans in ATB as compared to LTBI individuals represents a logical consequence of the broad difference in inflammatory state between the populations.

However, independent of general differences in proinflammatory glycosylation patterns between ATB and LTBI individuals, remarkably, IgG from LTBI patients additionally possessed less core fucosylation, linked to enhanced NK cell activating antibodies and significantly elevated FcγRIIIa binding compared to ATB patients (Lu et al. 2016). These unique LTBI-derived antibodies drove enhanced killing of intracellular *Mtb* in infected primary human macrophages compared with purified IgG from ATB individuals (Lu et al. 2016). Thus, the distinct Fc glycosylation patterns in LTBI individuals may be associated with enhanced *Mtb* control.

Collectively, studies in *Mtb* infection clearly point to similar IgG Fc-glycan changes in individuals with progressive, active disease to those reported during severe HBV infection, but they additionally reveal the presence of unique Fc-glycan profiles in individuals that control tuberculosis disease, which may actively contribute to enhanced microbial control of the pathogen.

### HIV

With a reported 36.9 million people living with human immunodeficiency virus (HIV) and approximately 1 million people dying of HIV-related causes in 2017 (WHO 2018c), HIV is among the greatest global threats to human health. In general, most HIV-infected individuals have a significantly higher proportion of bulk agalactosylated and asialylated antibodies as compared to healthy controls (Moore et al. 2005; Vadrevu et al. 2018), also observed in individuals with autoimmune diseases (Parekh et al. 1989; Tomana et al. 1992; Trbojević Akmačić et al. 2015; Decker et al. 2016), and the chronic infectious diseases described above (Rademacher et al. 1988; Parekh et al. 1989; Pilkington et al. 1995; Ho et al. 2015). However, unlike autoimmune diseases such as RA and HBV, where antibody glycosylation profiles normalize when a disease resolves, HIV-infected individuals that are treated with antiretroviral therapy (ART)—resulting in viral suppression—have been reported to retain abnormally high levels of IgG agalactosylation (Moore et al. 2005; Ackerman et al. 2013) and asialylation (Vadrevu et al. 2018), suggesting that B cells may be permanently altered by HIV infection. This permanent damage may be related to HIV persistence in germinal centers—even after ART initiation (Fukazawa et al. 2015)—that may perpetually result in perturbed B cell activation, and/or due to HIV persistence in the bone marrow (Carter et al. 2010), and thus perturbed de novo lymphocyte development.

Irreparable changes in fucosylation have also been reported, as HIV-negative individuals exhibited less IgG Fc fucosylation compared to HIV-positive unsuppressed and HIV-infected ART-suppressed individuals (Vadrevu et al. 2018). Furthermore, in successfully ART-suppressed patients, the levels of certain afucosylated IgG structures—G1, G2 and G2B—were noted to have a significant negative correlation with the frequency of HIV-infected cells, quantified by cell-associated DNA and RNA levels. These data point to the potential role of afucosylated IgG structures, known to harbor greater ADCC activity, in maintaining a smaller HIV cellular reservoir size during ART.

Although a majority of HIV-infected individuals are unable to control viral replication in the absence of ART and ultimately progress toward acquired immune deficiency syndrome (AIDS) if untreated (Deeks et al. 2015), approximately 1% of infected individuals, termed “spontaneous controllers,” are able to control infection spontaneously in the absence of therapy (Lambotte et al. 2005; Madec et al. 2005; Thèze et al. 2011). Antibody Fc-profiling highlighted the existence of significantly higher levels of agalactosylated antibodies in controllers compared to individuals with progressive infection (Ackerman et al. 2013). These exaggerated levels of agalactosylation in HIV controllers are likely a marker of the containment and persistence of HIV within germinal centers, despite systemic control of the infection. Indeed, the persistence of a viral reservoir for life at the site of B cell activation likely results in perpetual B cell activation and, therefore, the production of consistently elevated IgG agalactosylation, as alluded to above. However, interestingly, while not observed in bulk circulating antibodies, spontaneous controllers additionally elicited HIV-specific antibodies with a selective loss of fucosylated IgG Fc-glycan—G1F and G2F—linked to enhanced NK cell activation, as compared to progressive patients (Ackerman et al. 2013). These data point to both generalized inflammatory changes on bulk antibodies and selective changes in antigen-specific antibody glycosylation that track with enhanced NK cell activity in subjects with spontaneous control of HIV.

**Fig. 2:**
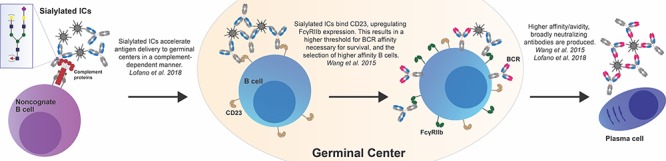
Potential roles for IgG Fc sialylation in driving the evolution of higher avidity and affinity antibody responses. Lofano et al. demonstrate that sialylated immune-complexes (ICs) accelerate antigen delivery to the germinal center in a complement-dependent manner, resulting in the generation of high avidity antibodies. While the pictured model implicates noncognate B cells in improved antigen deposition in the germinal center, other immune cell types expressing complement receptors, including subcapsular sinus macrophages, may also facilitate improved antigen delivery. Wang et al. demonstrate that within the germinal center, sialylated IgG antibodies bind CD23 present on B cells, increasing FcγRIIb surface expression and thus increasing the threshold for B cell receptor (BCR) signaling. This modulation of the germinal center reaction results in the generation of broadly neutralizing, high affinity antibodies.

Recently, additional changes in IgG Fc glycosylation have been noted in a subset of HIV-infected individuals that unfortunately progress to AIDS but naturally evolve broadly neutralizing antibodies that able to block a remarkable number of global viral strains (Lofano et al. 2018). While these neutralizing antibodies are unable to prevent progression in these individuals, defining the immune mechanisms that permit their evolution likely holds the key to vaccine development to block HIV at a global level. Specifically, analysis of HIV-specific antibody Fc profiles across individuals with broadly neutralizing antibody responses, and those without, highlighted the presence of an enrichment of sialylated Fc-glycans in neutralizers (Lofano et al. 2018). Strikingly, mechanistic studies aimed at defining the potential functional role of sialylation demonstrated enhanced antibody titers, affinity maturation, B cell numbers and antigen deposition in germinal centers in mice immunized with sialylated HIV antigen containing HIV-specific immune complexes. Enhanced affinity maturation driven by sialylated immune-complexes was also found to be complement-dependent, highlighting a novel axis by which sialylation may enhance antigen capture, presentation, and/or retention within germinal centers (Lofano et al. 2018). Overall, these data suggest a role for IgG sialylation in antigen delivery to the lymph node, resulting in improved germinal center activity, ultimately pointing to a noncanonical function of antibody glycosylation in the modulation of immunity, rather than in direct antipathogen activity (Figure 2).

Thus, in the context of HIV infection, discrete changes in the variably added glycans have been noted across patient populations, highlighting the remarkable plasticity of antibody glycosylation, and the ability for directed IgG Fc glycosylation to shape immunity.

### Influenza

Influenza virus is the causative agent of influenza, an acute respiratory infection resulting in an estimated 4 million severe cases and approximately 290,000—650,000 deaths annually (WHO 2018d). Influenza-specific antibodies exhibit remarkably different IgG Fc glycosylation profiles compared to HIV-specific antibodies profiled from the same individuals, pointing to differences in antibody glycan profiles across antigen specificities (Mahan et al. 2016). Specifically, influenza-specific antibodies had increased galactosylation and sialylation, but less bisection compared to both HIV-specific and bulk circulating antibodies (Mahan et al. 2016). Given that influenza hemagglutinin (HA) binds to sialic acid to facilitate viral invasion of host cells (Krammer et al. 2018), it is possible that the virus may select for an enrichment of sialylated glycans. However, this glycan profile may also represent a programed Fc-glycan profile distinct to influenza-specific immunity.

Furthermore, a study that examined IgG Fc glycosylation after influenza vaccination found that while vaccination did not impact the bulk IgG glycosylation profile of patients, anti-influenza IgG exhibited even further increased levels of galactosylation and sialylation following vaccination (Selman et al. 2012). Given the lack of virus or appreciable levels of HA, this result argues for a selective vaccine-induced shift in antigen-specific Fc glycosylation. A more recent study not only corroborated these results but also found an intriguing relationship between preexisting anti-influenza IgG Fc glycosylation and the immune response to influenza vaccination (Wang et al. 2015). Specifically, individuals with the highest levels of influenza HA-specific IgG sialylation 1 week post-vaccination were observed to generate higher affinity and enhanced hemagglutinating antibodies 3 weeks post-vaccination (Wang et al. 2015). Enhanced affinity maturation by sialylated immune complex-driven immunization was shown to drive the upregulation of the inhibitory FcγRIIb on B cells, in a CD23-dependent manner in vivo in mice, enhancing the threshold of activation of B cell receptors (Wang et al. 2015). Consistent with these observations, vaccination of mice with sialylated immune complexes drove higher affinity IgG (Wang et al. 2015), which resulted in enhanced protection across distinct strains of influenza virus (Wang et al. 2015). Thus, similar to HIV infection, in influenza, IgG Fc sialylation contributes to the improved maturation of the humoral immune response by inducing higher affinity antibodies with neutralizing potential that able to drive protection from infection (Figure 2; Wang et al. 2015).

Collectively, studies on influenza infection and vaccination clearly point to a unique glycan profile on influenza-specific antibodies compared to those elicited against other antigens, as well as to a critical role for sialylation of influenza-specific antibodies in shaping overall influenza specific humoral immunity.

### Dengue

Dengue virus (DENV) is a mosquito-borne pathogen that is widespread in countries with tropical and subtropical climates, though its global incidence continues to increase globally (WHO 2019b). DENV infects an estimated 390 million people annually, of which approximately 96 million cases manifest clinically (WHO 2019b). There are four distinct serotypes of DENV, and while initial DENV infection by any of the four DENV serotypes generally results in flu-like symptoms (WHO 2019b), reinfection with a heterologous serotype of the virus may lead to the development of more severe disease, termed dengue hemorrhagic fever (DHF; Katzelnick et al. 2017). DHF is believed to be caused by antibody-dependent enhancement (ADE) (Katzelnick et al. 2017). More specifically, the model suggests that at subneutralizing antibody concentrations, antibodies with poor cross-neutralizing activity to the new strain enhance, rather than block, infection (Katzelnick et al. 2017). However, while reinfection with a different serotype of DENV represents the greatest risk factor for the development of DHF, this event still only occurs in approximately 15% of reinfection cases, as such, the host determinants that define susceptibility for DHF remain poorly defined (Wang et al. 2017).

Interestingly, glycosylation of monoclonal antibodies has been shown to regulate ADE activity in vitro (Dent et al. 2016; Injampa et al. 2017). Specifically, comparison of ADE across wild-type or N297Q Fc-mutant IgG demonstrated that only the wild-type IgG1 was able to drive ADE (Injampa et al. 2017). The lack of ADE in the setting of the aglycosylated IgG clearly demonstrated the importance of Fc:FcR interactions in driving infection (Injampa et al. 2017). Further analysis of the importance of the glycan itself in ADE was assessed using monoclonals generated in plant and mammalian cells (Dent et al. 2016). Specifically, a DENV-specific monoclonal antibody was produced in engineered plants, *Nicotiana benthamiana*, which generate a highly homogenous antibody *N*-glycan profile with only the core antibody Fc-glycan (lacking each of the variably added glycan moieties—no fucose, galactose, sialic acid or b-GlcNAc), as well as in Chinese hamster ovary (CHO) cells, which generate more heterogeneous glycoforms (Dent et al. 2016). Interestingly, despite the presence of identical F(ab′)_2_ domains, differences were observed in ADE across the two Fc-glycan variants. Specifically, unlike the DENV-specific antibody variant produced in CHO cells, the plant produced antibody variant did not induce ADE (Dent et al. 2016). These data suggest that the addition of one of the variably added carbohydrates is likely key to enhanced ADE in vitro.

Recent work has shed additional light on the specific glycan changes that may play a role in severe DENV disease in vivo (Wang et al. 2017). Isotype/subclass-specific glycan profiling in individuals reinfected with DENV highlighted the presence of increased levels of afucosylated IgG1 specific to DENV in patients with more severe DENV disease (Wang et al. 2017). Interestingly, the same shift in afucosylation was also observed in influenza-specific antibodies, pointing to a global shift in fucosylation (Wang et al. 2017). Given the knowledge that anti-DENV antibodies can cross-react with platelet antigens (Sun et al. 2007), as well as the observed correlation between afucosylated IgG levels and thrombocytopenia, the authors hypothesized that anti-DENV IgGs that cross-react with platelet antigens may drive platelet loss and consequently more severe DENV disease (Wang et al. 2017). Strikingly, the administration of IgG from patients with and without thrombocytopenia in human FcR knockin mice caused a significant drop in platelet counts in mice receiving IgG from the thrombocytopenia patients in an FcγRIIa- and FcγRIIIa-dependent manner (Wang et al. 2017). Furthermore, the removal of the IgG glycan resulted in decreased platelet destruction (Wang et al. 2017). While additional studies should be performed to define the exact mechanism driving global DHF, this study has critically demonstrated that a subset of individuals respond to DENV infection by producing high levels of afucosylated IgG, which likely drive thrombocytopenia and enhanced disease, in vivo.

Thus, work performed on DENV infection point to the critical role for antibody glycosylation in the pathogenesis of disease both in the cellular enhancement of infection in vitro and in the platelet destruction in vivo.

### Ebola

Ebola virus (EBOV) is a rare, but often fatal disease in humans if left untreated (WHO 2018b). Case fatality rates have ranged from 25 to 90% depending on the strain and population (WHO 2018b). Since EBOV disease was first reported in 1976, there have been regular outbreaks in several African countries—including the 2014–2016 epidemic in West Africa that ended with more than 28,600 cases and 11,325 deaths—highlighting the need for novel vaccines and therapeutics to limit the spread of disease (Kaner and Schaack 2016).

Studies on EBOV survivors, vaccines and from monoclonal therapeutic design efforts have all collectively demonstrated the importance of antibodies in the control and clearance of EBOV (Zeitlin et al. 2011; Olinger et al. 2012; Saphire, Schendel, Fusco, et al. 2018a; Saphire, Schendel, Gunn, et al. 2018b). Furthermore, multiple studies have demonstrated the critical importance of Fc glycosylation in in vivo protection (Zeitlin et al. 2011; Olinger et al. 2012). Specifically, an EBOV-specific monoclonal antibody expressed in either glyco-engineered plants (*N. benthamiana*) or CHO cells, as well as an N297Q Fc-variant were generated and tested for protective efficacy against lethal EBOV challenge in mice (Zeitlin et al. 2011). Interestingly, the *N. benthamiana-*derived antibody carrying only the core glycan most potently prolonged mouse survival compared to other antibody variants (Zeitlin et al. 2011). Moreover, administration of a cocktail of three EBOV-specific monoclonal antibodies, produced in either *N. benthamiana* or CHO cells, to rhesus macaques, showed a similar enhanced protection by the plant-derived antibodies (Olinger et al. 2012). Specifically, though the sample sizes were small, 100% of primates receiving the plant-derived antibody cocktail survived, as compared to 50% of primates receiving the CHO-derived antibody cocktail, despite the fact that 3-fold less of the plant-derived antibody pool was administered (Olinger et al. 2012). Collectively, due to the afucosylated nature of the antibodies produced in the plant system (Zeitlin et al. 2011; Olinger et al. 2012), the authors speculated that fucose rather than galactose or sialic acid differences were likely responsible for protection via enhancing ADCC activity.

Furthermore, dissection of the humoral- and Fc-glycan-dependent correlates of protection against EBOV in mice was recently performed using a library of 168 EBOV-specific monoclonal antibodies (Gunn et al. 2018; Saphire, Schendel, Fusco, et al. 2018a; Saphire, Schendel, Gunn, et al. 2018). While neutralization capacity was found to be strongly associated with protection in mice, neutralization alone was not sufficient to explain all protection. Indeed, consistent with a protective role for antibodies that drive ADCC in the nonhuman primate model of EBOV infection, afucosylated and bisected IgG Fc-glycan structures—G1, G2S1B and G2S1FB—were linked to protection (Gunn et al. 2018). Moreover, monocyte and neutrophil phagocytosis represented additional correlates of protection, implicating alternate Fc-glycan-dependent innate immune effector functions, beyond ADCC, in protection against this lethal pathogen (Gunn et al. 2018).

Thus, in the context of EBOV infection, studies across mice and nonhuman primates clearly demonstrate a critical role for Fc-glycans in shaping protective immunity.

## Discussion

Although the impact of IgG Fc glycosylation on only a few viral and bacterial diseases were described as models, they reveal a few key cross-pathogen themes related to how antibody glycosylation may be impacted by and ultimately shape the immune response to infection.

Firstly, evidence unequivocally indicates that distinct glycosylation changes occur on antibodies in an antigen-specific manner. For example, HIV-specific antibodies were reported to have remarkably different IgG Fc-glycan patterns than influenza-specific antibodies from the same individuals (Mahan et al. 2016). Similarly, in the setting of vaccination, while the bulk IgG glycosylation profile of patients was not significantly altered, anti-influenza and anti-tetanus IgG exhibited increased levels of sialylation following vaccination (Selman et al. 2012).

**Fig. 3:**
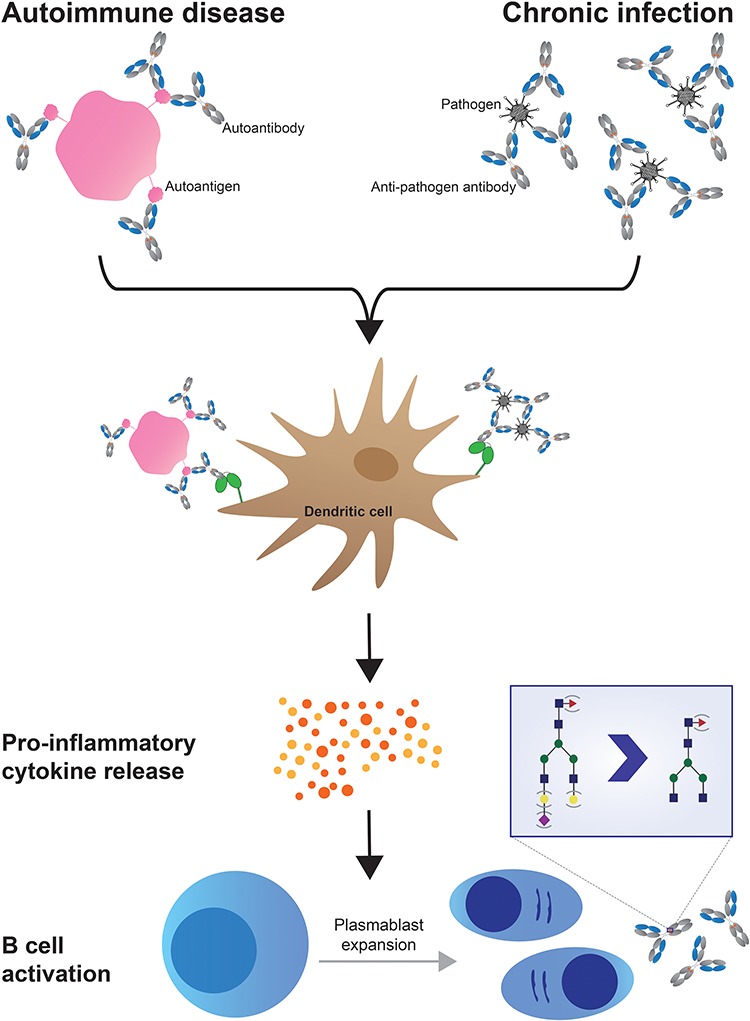
Potential model by which autoimmune diseases and chronic infections drive inflammatory IgG release. Persistent antigen exposure results in the formation of immune complexes that drive Fcγ receptor-mediated immune activation and proinflammatory cytokine release. Inflammation may then precipitate the conversion of B cells into antibody-secreting plasmablasts tuned to release largely agalactosylated, asialylated IgG, causing a shift to the inflammatory IgG Fc glycosylation profile observed in numerous autoimmune and chronic infectious diseases.

Furthermore, clear parallels exist between antibody glycosylation in autoimmune disease and chronic infectious disease, both of which are marked by high, protracted inflammation (Rademacher et al. 1988; Parekh et al. 1989; Moore et al. 2005; Vadrevu et al. 2018). Chronic manifestations of HBV, tuberculosis and HIV infection each exhibit autoimmune like profiles, marked by similar glycan shifts, most notably, in the accumulation of high levels of agalactosylation and asialylation (Rademacher et al. 1988; Parekh et al. 1989; Moore et al. 2005; Vadrevu et al. 2018). These data raise the possibility that chronic inflammation across infectious and noninfectious diseases may lead to the same agalactosylated IgG Fc glycosylation profile. Indeed, chronic pathogen replication and antigenic stimulation likely drive perpetual immune activation, proinflammatory cytokine release and B cell activation, resulting in the production of consistently high levels of inflamed agalactosylated IgG (Figure 3). Thus, it is likely that in the case autoimmunity and select infectious diseases, the skew toward agalactosylated and asialylated IgG is a consequence of similar inflamed immunologic pathways and thus represents a humoral marker of inflammation.

Additional analyses across disease progression spectra provide enhanced resolution of discrete Fc-glycan modifications that vary with disease state. In the case of HBV, tuberculosis, HIV and DENV infection, various shifts in IgG Fc glycosylation correspond with disease severity (Ackerman et al. 2013; Lu et al. 2016; Wang et al. 2017), potentially providing diagnostic and perhaps even prognostic value. Notably, in the case of *Mtb* infection, where traditional immune-based diagnostics have failed to distinguish controlled LTBI from uncontrolled ATB (Pai et al. 2016), differences in IgG Fc-glycan profiles could separate the two groups (Lu et al. 2016), pointing to the potential utility of antibody glycosylation-based diagnostics for the resolution of tuberculosis disease.

Critically, beyond marking differences in disease severity, evidence indicates that antibody glycosylation can functionally improve the immune response to infection (Ackerman et al. 2013; Lu et al. 2016). For example, in tuberculosis, LTBI individuals generated higher levels of afucosylated antibodies, with increased FcγRIIIa binding, as well as an increased capacity to kill intracellular *Mtb* (Lu et al. 2016). Similarly, individuals who control HIV also generated enhanced levels of HIV-specific afucosylated antibodies that track selectively with enhanced antiviral activity (Ackerman et al. 2013), a modification that was also noted to protect against EBOV (Zeitlin et al. 2011; Olinger et al. 2012). Further, work within the field of HIV infection and influenza vaccination has revealed a noncanonical role for sialylated IgG in driving the evolution of a higher avidity and affinity antibody responses to control pathogens more effectively (Selman et al. 2012; Wang et al. 2015; Lofano et al. 2018). Thus, in this context, antigen-specific antibody glycan shifts can have both direct and indirect effects on antipathogen immunity.

Conversely, in the context of DENV infection, a select pattern in IgG glycosylation—marked by a generalized loss of fucose—was noted to associate with enhanced disease severity (Wang et al. 2017). This result strongly argues for a critical need to carefully control Fc-glycosylation in an antigen-specific manner. Moreover, it provides a cautionary warning that universal glycan modifications are unlikely to translate to microbial control across all pathogens. Ultimately, divergent roles for specific glycan patterns in different infections likely arise due to differences in mode of infection, niche of infection (intra- versus extracellular), evasive mechanisms, cellular tropism, etc., all likely requiring distinct innate immune effector functions to drive control and clearance of infection. Thus, future work across infectious models are needed to provide enhanced resolution of the natural immune glycan modifications that track with the control of different pathogens.

## Future perspectives

Collectively, the work described above demonstrate mechanisms by which antibody Fc glycosylation can direct the immune response to drive microbial control, pointing to the therapeutic potential for antibodies that harness Fc glycosylation to combat infectious diseases. Thus, as antibody-based therapeutics continue to expand beyond the fields of cancer and autoimmunity and into the field of infectious disease, identifying antibody glycosylation profiles that track with and drive microbial control of different pathogens will be critical to ultimately engineering therapeutic antibodies that optimally drive pathogen elimination. Furthermore, efforts to engineer antibodies with any of the 36 theoretical IgG Fc-glycan structures are underway (Dekkers et al. 2016; Mimura et al. 2018), which should allow Fc-glycan-optimized antibodies to be therapeutically exploited once the target antibody Fc profile to combat the pathogen of interest is identified.

In addition to the potential to leverage antibody glycosylation in a therapeutic setting, evidence indicates that antibody glycosylation can be modified by vaccination (Selman et al. 2012; Vestrheim et al. 2014; Wang et al. 2015; Mahan et al. 2016). As described above, sharp increases in anti-influenza- and anti-tetanus-specific IgG galactosylation and sialylation are observed following influenza and tetanus vaccination respectively (Selman et al. 2012). A similar increase in IgG Fc galactosylation and sialylation was additionally reported following pneumococcal and meningococcal vaccination (Vestrheim et al. 2014). Furthermore, in an experimental HIV vaccine (Mahan et al. 2016), where vaccinated individuals were observed to exhibit striking bulk IgG glycosylation differences between geographically distinct vaccination sites, vaccine-specific patterns were remarkably similar following immunization across all vaccinees (Mahan et al. 2016). This argues that glycosylation can be tuned by vaccination in a manner independent of baseline differences in antibody glycosylation. Nevertheless, while the potential for leveraging vaccination to direct antibody glycosylation and improve vaccine efficacy is an exciting prospect, in humans, the way in which vaccine parameters such as adjuvant, boosting strategy and administration route precisely impact antibody Fc glycosylation remains unclear. Thus, learning how to direct, control and maintain target Fc-glycan profiles of interest may provide an alternate means by which next-generation vaccines may drive enhanced control over infections.

## Concluding remarks

Ultimately, infectious diseases highlight the clear role of the immune system in shaping and controlling Fc glycosylation. Given the delicate balance of protective versus pathological Fc profiles across infectious diseases, future efforts focused on defining antibody Fc profiles that track with disease outcomes may provide critical insights related to the target product profiles associated with protective immunity for next-generation therapeutic and vaccine designs.

## Funding

GPiB National Institutes of Health (T32AI132120).

## Conflict of interest statement

None declared.

## Abbreviations

AIDS, acquired immune deficiency syndrome; ADE, antibody-dependent enhancement; ATB, active tuberculosis disease; CHO, Chinese hamster ovary; DENV, Dengue virus; DHF, dengue hemorrhagic fever; EBOV, Ebola virus; FcRs, Fc receptors; GlcNAc, *N*-acetylglucosamine; HA, hemagglutinin; HIV, human immunodeficiency virus; IgG, immunoglobulin G; LTBI, controlled latent tuberculosis infection.
